# Insulin use and Excess Fracture Risk in Patients with Type 2 Diabetes: A Propensity-Matched cohort analysis

**DOI:** 10.1038/s41598-017-03748-z

**Published:** 2017-06-19

**Authors:** Eladio Losada-Grande, Samuel Hawley, Berta Soldevila, Daniel Martinez-Laguna, Xavier Nogues, Adolfo Diez-Perez, Manel Puig-Domingo, Dídac Mauricio, Daniel Prieto-Alhambra

**Affiliations:** 1 Department of Medicine, Autonomous University of Barcelona, Barcelona, Spain; 2 Endocrinology Section, Internal Medicine Department, Hospital Can Misses, Ibiza, Spain; 3 Musculoskeletal Pharmaco- and Device Epidemiology, Centre for Statistics in Medicine, Nuffield Department of Orthopaedics, Rheumatology and Musculoskeletal Sciences, University of Oxford, Oxford, UK; 4 Department of Endocrinology and Nutrition, University Hospital & Health Sciences Research Institute “Germans Trias i Pujol”, Badalona, Spain; 5 CIBER of Diabetes and Associated Metabolic Diseases (CIBERDEM), Instituto de Salud Carlos III, Majadahonda, Spain; 6 GREMPAL Research Group, IDIAP Jordi Gol Primary Care Research Institute, Autonomous University of Barcelona, Barcelona, Spain; 7 Internal Medicine Department, IMIM (Hospital del Mar Research Institute), Autonomous University of Barcelona, Barcelona, Spain; 8 CIBER of Healthy Ageing and Frailty Research (CIBERFes), Instituto de Salud Carlos III, Majadahonda, Spain

## Abstract

Despite normal to high bone mineral density, patients with type 2 diabetes (T2DM) have an increased fracture risk. T2DM medications could partially account for this excess risk. The aim of this study was to assess the association between insulin use and bone fracture risk in T2DM patients. A population-based matched cohort study based on a primary care records database validated for research use (Catalonia, Spain) was performed. Propensity score (PS) for insulin use was calculated using logistic regression including predefined predictors of fractures. A total of 2,979 insulin users and 14,895 non-users were observed for a median of 1.42 and 4.58 years respectively. Major fracture rates were 11.2/1,000 person-years for insulin users, compared with 8.3/1,000 among non-users. Matched models confirmed a significant association, with an adjusted subhazard ratio (adj SHR) of 1.38 [95% CI 1.06 to 1.80] for major fractures. No differences between types of insulin or different regimens were found. Estimated number needed to harm (fracture) was 82 (95% CI 32 to 416). Insulin use appears to be associated with a 38% excess fracture risk among T2DM patients in the early stages of the disease. Fracture risk should be included among the considerations to initiate insulin treatment.

## Introduction

Patients with type 2 diabetes mellitus (T2DM) have an increased risk of bone fractures^1–13^ despite a normal to high bone mineral density (BMD) compared with non-diabetic subjects^10, 14–17^. Although the mechanisms underlying this observed excess fracture risk among T2DM patients remain unclear, some evidence indicates that fragility fractures in T2DM patients may be explained by the presence of impaired structural properties that compromise bone quality and ultimately lead to bone fragility^18, 19^.

Risk factors associated with fracture risk in T2DM include duration of disease^7, 9, 14^, diabetic complications [impaired vision^1, 14^, peripheral neuropathy^20^, orthostatic hypotension^21^, etc.], episodes of hypoglycaemia^22^, increased risk of falling^20, 22–24^, inadequate glycaemic control^25^, and some antidiabetic medications that appear to affect bone metabolism, such as glitazones^26, 27^. Conversely, data on the impact of insulin treatment on fractures in T2DM are scarce and remain controversial, with some^4, 6–9, 13^ but not all^1, 28^ studies showing an increased risk of fracture. Most of the previous cohorts had no information on date of diagnosis or of insulin therapy initiation, making it difficult for researchers to accurately estimate attributable excess risk^1, 4, 6–9, 13^.

The increased risk observed in most observational studies has also been attributed to disease severity as most of the previously analysed cohorts lacked information on glycated haemoglobin (HbA1c), a key parameter to assess T2DM metabolic control^1, 4–9, 14^. However, insulin therapy could affect fracture risk in several ways: it is related to an increased risk of hypoglycaemia which may induce falls and, in some experimental models, it has been associated with a disregulation of osteoclastogenesis^29, 30^. Nonetheless, no direct negative effect of insulin therapy on bone has yet been directly demonstrated in randomised controlled trials.

In the present study, we analysed detailed clinical information from a large cohort of newly diagnosed T2DM patients to investigate an association between insulin use and fracture risk in these subjects.

## Results

We identified 53,853 patients newly diagnosed with T2DM who fulfilled inclusion and exclusion criteria in the Information System for Research Development in Primary Care database (Catalan acronym, SIDIAP) between 2006 and 2013. Of these, 3,227 initiated insulin therapy (with ≥2 months persistence) and 50,626 (94%) never used insulin throughout the study period. Baseline characteristics differed significantly between insulin users and non-users in terms of: age, body mass index (BMI), socio-economic status (SES), smoking status, alcohol intake, previous falls, glomerular filtration rate (GFR), neuropathy, HbA1c level, use of medications related with fracture risk (diuretics, antihypertensives, diuretics plus antihypertensives, and steroids), use of some antidiabetic medications (metformin, sulphonylureas, and meglitinides), and index year of diagnosis. Most of these differences were attenuated after propensity score (PS) matching: 2,979/3,227 (92.3%) insulin users were matched to 14,895/50,626 (29.4%) non-users of similar characteristics. Differences (P < 0.1) remained post-matching only in terms of BMI and use of certain medications (antihypertensives, steroids, and meglitinides) (Table 1).

**Table 1:** Baseline characteristics of patients with incident T2DM stratified by insulin use vs non-insulin use during follow-up.

	Unmatched Participants					Matched Participants				
	Non-users of Insulin (N = 50,626)		Users of Insulin (N = 3,227)		P-value**	Non-users of Insulin (N = 14,895)		Users of Insulin (N = 2,979)		P-value**
	**N**	**%**	**N**	**%**		**N**	**%**	**N**	**%**	
**Demographic risk factors**										
Sex, male	28,323	60	1,844	57.1	0.18	8,411	56.5	1,678	56.3	0.89
Age, years										
Mean (SD)	63.7 (11.2)		61.2 (11.9)		<0.001	61.7 (11.9)		61.8 (11.9)		0.76
Body Mass Index §^∧^										
Mean (SD)	31.1 (5.2)		30.8 (5.9)		0.002	31.0 (5.6)		30.8 (5.8)		0.097
Socio-economic status ±										
Quintile1 (−1.83 to 0.03)	9,379	18.5	581	18.0		2,654	17.8	532	17.9	
Quintile 2 (0.03 to 0.50)	9,387	18.5	557	17.3		2,589	17.4	518	17.4	
Quintile 3 (0.50 to 0.94)	9,368	18.5	582	18.0		2,762	18.5	536	18.0	
Quintile 4 (0.94 to 1.52)	9,347	18.5	541	16.8		2,561	17.2	513	17.2	
Quintile 5 (1.52 to 5.28)	9,238	18.3	664	20.6		2,975	20.0	599	20.1	
Unknown	3,907	7.7	302	9.4	<0.001	1,354	9.1	281	9.4	0.98
Smoking										
Never	18,824	37.2	1,005	31.1		4,760	32.0	938	31.5	
Ex-smoker	6,914	13.7	551	17.1		2,438	16.4	485	16.3	
Current	7,737	15.3	441	13.7		2,035	13.7	416	14.0	
Unknown	17,151	33.9	1,230	38.1	<0.001	5,662	38	1,140	38.3	0.94
Drinking										
Never	21,325	42.1	1,149	35.6		5,450	36.6	1,128	37.9	
Moderate	10,777	21.3	341	10.6		1,663	11.2	337	11.3	
Severe	1,458	2.9	66	2.1		329	2.2	63	2.1	
Unknown	17,066	33.7	1,671	34.8	<0.001	7,453	50.0	1,451	48.7	0.55
**Conditions affecting fracture risk**										
Fracture	1,053	2.1	57	1.8	0.22	257	1.7	54	1.8	0.74
Stroke	1,561	3.1	113	3.5	0.18	508	3.4	108	3.6	0.56
Myocardial infarction	1,463	2.9	95	2.9	0.86	409	2.8	88	3.0	0.53
Falls	456	0.9	16	0.5	0.017	76	0.5	16	0.5	0.85
**Conditions related to diabetes**										
eGFR										
<60	6,043	11.9	409	12.7		1,885	12.7	375	12.6	
Unknown	8,326	16.5	834	25.8	<0.001	3,842	25.8	767	25.8	0.99
Neuropathy	692	1.4	181	5.6	<0.001	530	3.6	108	3.6	0.22
HbA1c±^∧^										
Quintile 1 (3.5 to 5.7)	9,285	18.3	320	9.9		1,564	10.5	318	10.7	
Quintile 2 (5.8 to 6.2)	9,270	18.3	267	8.3		1,340	9.0	267	9.0	
Quintile 3 (6.3 to 6.6)	7,489	14.8	247	7.7		1,235	8.3	243	8.2	
Quintile 4 (6.6 to 7.3)	7,328	14.5	355	11.0		1,738	11.7	348	11.7	
Quintile 5 (7.4 to 19.1)	7,147	14.1	1,046	32.4		4,285	28.8	881	29.6	
Unknown	10,107	20.0	992	30.7	<0.001	4,733	31.8	922	31.0	0.94
**Medications associated with fracture risk**										
Diuretics	6,727	13.3	351	10.9	<0.001	1,743	11.7	330	11.1	0.33
Antihypertensive	23,035	45.5	1,729	53.6	<0.001	6,488	43.6	1,610	54.0	<0.001
Diuretic plus antihypertensive	12,732	25.2	725	22.5	0.001	3,436	23.1	669	22.5	0.47
Steroids	3,163	6.3	346	10.7	<0.001	1,461	9.8	322	10.8	0.096
**Medications associated with osteoporosis treatment**										
Bisphosphonates	2,207	4.4	130	4.0	0.37	565	3.8	122	4.1	0.43
SERMs	295	0.6	14	0.4	0.27	59	0.4	13	0.44	0.75
Teriparatide	44	0.1	1	<0.1	0.51	4	<0.1	1	<0.1	0.84
**Medications for diabetes treatment**										
Metformin***	16,690	33.0	901	27.9	<0.001	4,203	28.2	839	28.2	0.95
Sulphonylureas	1,691	3.3	272	8.4	<0.001	1,005	6.8	215	7.2	0.35
Meglitinides	227	0.5	47	1.5	<0.001	153	1.0	41	1.4	0.093
GLP-1 analogues	13	<0.1	1	<0.1	0.86	5	<0.1	1	<0.1	1
DPP4-i	129	0.25	13	0.4	0.11	60	0.4	13	0.44	0.79
Other (non-insulin) meds	73	0.14	19	0.6	<0.001	54	0.36	15	0.5	0.26
Index year****										
2006	6,276	12.4	823	25.5		3,453	23.2	627	21.1	
2007	6,394	12.6	649	20.1		2,977	20.0	606	20.3	
2008	6,503	12.9	550	17.0		2,618	17.6	541	18.2	
2009	7,598	15.0	453	14.0		2,211	14.8	453	15.2	
2010	8,355	16.5	354	11.0		1,723	11.6	354	11.9	
2011	7,674	15.2	235	7.3		1,147	7.7	235	7.9	
2012	7,826	15.5	163	5.1	<0.001	766	5.1	163	5.5	0.35

^§^Although presented here as continuous; BMI and age were entered into PS model as categorical variables incorporating a missing category.

**P-values are reported for chi-square (categorical variables) and t-test (continuous variables), comparing the distribution of baseline characteristics between insulin users and non-users.

***Metformin counted in year prior to index date (other comedications only counted in prior month).

****Index date defined as 01/01/2006 or date of T2DM diagnosis (if after 01/01/2006) or date of enrolment with GP (if after T2DM diagnosis). This was ‘baseline’, and a time-varying covariates model was used, splitting follow-up time at start of insulin use.

^∧^Missingness: Non-matched [BMI = 16.0% (non-insulin user), 23.9% (insulin user); HbA1c = 19.7% (non-insulin user), 30.7% (insulin user)]; matched [BMI = 23.8% (non-insulin user), 23.4% (insulin user); HbA1c = 31.8% (non-insulin user), 31.0% (non-insulin user). ^±^Mean values within each quintile for the propensity score matched cohorts for socio-economic status: Q1 = −0.14, Q2 = 0.28, Q3 = 0.73, Q4 = 1.21, Q5 = 2.25 (non-user) and Q1 = −0.38, Q2 = 0.23, Q3 = 0.74, Q4 = 1.20, Q5 = 2.28; for HbA1c: Q1 = 5.32, Q2 = 6.01, Q3 = 6.44, Q4 = 6.95, Q5 = 8.88 (non-user) and Q1 = 5.27, Q2 = 6.00, Q3 = 6.45, Q4 = 9.97, Q5 = 9.66 (user).

Abbreviations: Type 2 diabetes mellitus (T2DM), estimated glomerular filtration rate (eGFR), haemoglobin A_1C_ (HbA1c), selective oestrogen receptor modulators (SERMs), glucagon-like peptide −1 (GLP-1), dipeptidyl peptidase-4 inhibitors (DPP4-i).

The included participants were then observed for a median inter-quartile range (IQR) of 4.84 (3.09 to 6.44) years, totalling 81,761 person-years (PYs). At least one major fracture occurred in 60/2979 insulin users (in ~5400 PYs) and 631/14,895 PS-matched non-users (in ~76,000 PYs), an equivalent to incidence rates of 11.2/1,000 (95% CI 8.7 to 14.4) and 8.3/1,000 PY (95% CI 7.6 to 8.9), respectively.

**Figure 1:**
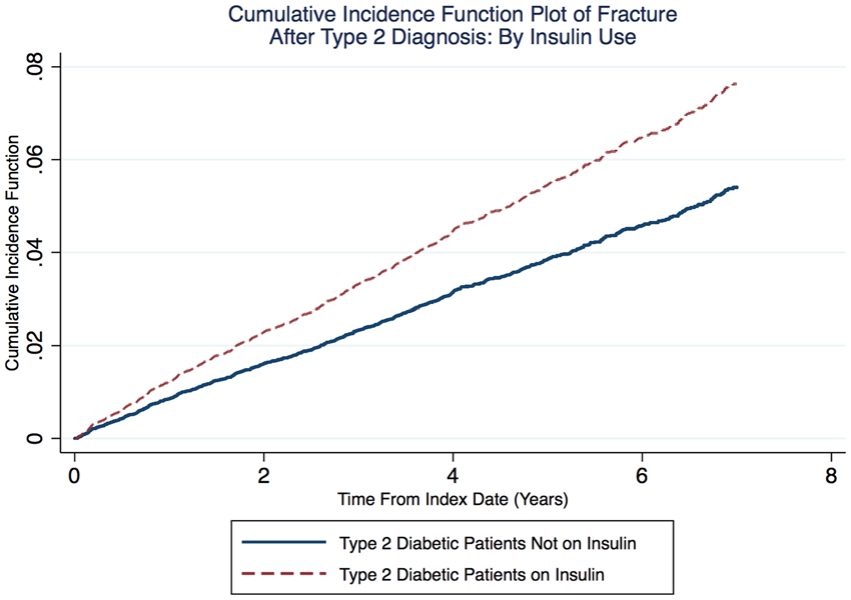
Cumulative incidence function plot of bone fracture after type 2 diabetes mellitus diagnosis between non-insulin and insulin users accounting for time-varying exposure.

Survival models confirmed an association between insulin use and fracture risk in the matched cohorts [unadjusted subhazard ratio (SHR) and (95% CI) 1.43 (1.10 to 1.86)], which remained unchanged after further adjustment for BMI, steroid use, meglitinides, and anti-hypertensive drugs (i.e. the variables that remained imbalanced after PS matching [P < 0.1]): adjusted (adj) SHR 1.38 (1.06 to 1.80) (Table 2). Fracture cumulative incidence function plots stratified by insulin use are depicted in Fig. 1.

**Table 2:** Fracture incidence among matched users of any insulin (N = 2,979) and non-users of insulin (N = 14,895).

Non-users of insulin (14,895)			Users of insulin (2,979)			SHR	adjSHR
N	Median follow-up (years) (IQR)	Rate (per 1000 PYs)	N	Median follow- up (years) (IQR)	Rate (per 1000 PYs)		
631	4.58 (2.60–6.32)	8.26 (7.64–8.93)	60	1.42 (0.67–2.67)	11.19 (8.69–14.42)	1.43 (1.10–1.86)	1.38 (1.06–1.80)

Only the first continuous insulin use considered (switching types was accounted for), then censored. All analyses include incident insulin use as a time-dependent covariate. Analyses are PS matched at a 5:1 ratio of non-users to users. Adjusted analyses control for outstanding confounders (P < 0.1), i.e. BMI, steroids, meglitinides, and anti-hypertensive therapy.

Abbreviations: N (number of fractures), Interquartile range (IQR), person-years (PYs), subhazard ratio (SHR), adjusted (adj).

At 5 years, the estimated number needed to harm (NNH) - i.e., the number of patients treated with insulin for 5 years needed to cause 1 major fracture – was 82 (95% CI 32 to 416).

Models assessing the association between insulin therapy adherence and fracture risk showed a dose-response gradient for the first three quartiles of medication possession ratio (MPR): adj SHR 2.58 (1.56 to 4.26) for those in the third quartile, compared with an adj SHR of 0.92 (0.50 to 1.71) for low-dose (first quartile) insulin users (Table 3). In a separate analysis to study the effect of different insulin types, 34 patients fractured while taking long-acting human insulin, compared to 12 fractures in patients treated with long-acting analogues (adj SHR 1.02 [0.52 to 1.99]); and 38 fractures were seen in users of long-acting compared to 5 users of mixed insulin regimens (short and fast-acting combination) (SHR 0.76 [0.30 to 1.96]) (data not shown).

**Table 3:** Fracture incidence among users of any insulin (N = 2,980) vs matched non-users (N = 24,900), stratified by quartiles of insulin medication possession ratio (MPR) within first year of first insulin use.

MPR		Non-users of insulin (14,900)			Users of insulin (2,980)			SHR	adj SHR
Quartile	Median (IQR)	N	Median follow-up (years)	Rate (per 1000 PYs)	N	Median follow-up (years)	Rate (per 1000 PYs)		
1	21 (21–31)	235	4.5	8.8	11	0.8	7.8	0.94 (0.51–1.73)	0.92 (0.50–1.71)
2	41 (41–51)	147	4.6	7.1	17	1.3	12.6	1.85 (1.12–3.04)	1.85 (1.12–3.05)
3	62 (62–72)	97	4.6	7.2	18	1.7	17.9	2.58 (1.56–4.26)	2.58 (1.56–4.26)
4	103 (84–123)	151	4.7	9.5	10	1.9	7.5	0.82 (0.43–1.57)	0.84 (0.45–1.60)

Only the first insulin use considered (i.e. switching types was not accounted for), then censored. All analyses include incident insulin use as a time dependent covariate. Analyses are PS matched, on a 5:1 ratio of non-users to users in a separate model from the main analysis. Adjusted analyses control for outstanding confounders (P < 0.1) and for propensity score.

Abbreviations: N (number of fractures), Interquartile range (IQR), person-years (PYs), subhazard ratio (SHR), adjusted (adj).

A number of sensitivity analyses were conducted to confirm the robustness of our findings. Firstly, we studied interactions with sex, age, and HBA1c levels, obtaining results that were similar to those in the main analysis. Results remained also unchanged after excluding early insulin users (and matched non-users) who initiated therapy within 6 months after T2DM diagnosis [adjusted SHR 2.00 (1.45 to 2.77)]; similarly, reclassifying insulin use in the first two months of treatment as non-user time obtained similar findings to our main analysis [adj SHR 1.28 (0.96 to 1.71)].

In an additional sensitivity analysis, results were unchanged when multiple imputation was used to account for missing BMI and HbA1c values (Supplementary Table 1). Also, further adjustment for median HbA1c over time after T2DM diagnosis did not modify the observed association (Supplementary Table 2). Finally, we conducted an analysis where insulin dosage (in the form of MPR) was corrected for BMI and stratified by gender. This confirmed our previous findings both in men (Supplementary Table 3) and women (Supplementary Table 4).

## Discussion

We report a significant (38%) excess risk of major osteoporotic fractures among recently diagnosed T2DM patients exposed to insulin therapy, compared with PS-matched insulin non-users with T2DM. This association remained unmodified after adjusting for imbalanced variables. Assuming a causal relationship and considering the estimated NNH, approximately 82 recently diagnosed T2DM would need to be treated with insulin to produce an attributable major fracture within 5 years. No differences were found in terms of excess fracture risk when users of different types of insulin (human vs. analogues, long-acting vs. long plus short-acting) were compared.

In general, our excess risk results were consistent with most published observational studies in which insulin therapy has been related to a higher risk of hip and major bone fractures^1, 13^. However, most of these studies investigated only subgroups of the population, specifically the elderly^7–9^ or postmenopausal women^4–6, 9^ and some did not differentiate between type 1 diabetes mellitus (T1DM) and T2DM^7, 13^. Additionally, most studies involved patients with pre-existing T2DM^6–9, 11, 13^ or did not report on disease duration^6^. Similarly, some previous reports lacked information on disease severity, as defined by glycaemic control^6–9, 11, 13^, and nearly all of the studies lacked information or did not adjust for the date of onset of insulin therapy, the type of insulin, or the regimen/dose used^6–9, 11–13^.

Conflicting data have been obtained when the effect of anti-diabetic treatments (including insulin) on bone fracture was studied as a primary endpoint. In a nationwide, population-based, case-control study in Denmark, insulin therapy showed a non-significant trend towards a decreased risk of bone fracture after adjusting for multiple confounders^11^. In an Italian case-control study^28^, insulin use during at least 36 months in the previous 10 years was not associated with fracture risk.

In our dataset, insulin use was associated with an increased risk of major osteoporotic fractures independent of T2DM disease duration, glycaemic control (disease severity), and other potential confounders. Therefore, our findings suggest that insulin therapy may have played a role in this excess risk rather than, as has been suggested by previous authors, acting as a surrogate of longer disease duration or severity^31, 32^.

Previous publications have indicated that insulin stimulates the regulation of osteoblast function, increasing the proliferation and differentiation from mesenchymal stem cells (MSC) in both animal models and human studies^33^. In rodents, including insulin receptor substrate-1 (IRS-1) and IRS-2 knock-out mice and streptozotocin-induced diabetic mice, studies have shown severe osteopenia, decreased bone formation, and severe growth retardation, respectively^34–36^. In humans, data from young T1DM patients show more osteopenia and osteoporosis than in non-diabetic subjects^37^. In a 7-year follow-up study in young patients with T1DM, treatment with intensive insulin therapy stabilized BMD and decreased bone resorption markers^38^.

In contrast to T1DM, where marked insulin deficiency is the pathogenetic substrate of hyperglycaemia, insulin resistance with relative insulin deficiency results in different degrees of hyperinsulinemia during much of the natural history of T2DM. Additionally, it is well known that once insulin treatment is established, adequate levels of portal insulin are obtained at the expense of peripheral hyperinsulinemia, which may have an impact on various organs and tissues (e.g., bone). *In vitro* and *in vivo* models have shown a relationship between hyperinsulinemia and alterations in bone resorption, as well as impaired bone formation via a negative regulation of osteoclastogenesis^29, 30^.

Long-acting human insulins are more closely related to symptomatic, overall, and nocturnal hypoglycaemias than are long-acting insulin analogues^39^. Short-acting insulins (basal-bolus or premixed insulin regimens) are most commonly used in more severe (less controlled) cases of T2DM, and are also associated with a higher risk of hypoglycaemia^40^. In our study, we did not find risk differences between users of different types of insulin. Participants taking higher doses of insulin had an increased fracture risk in a dose-response gradient for the 3 first quartiles; the absence of increased fractures in the fourth quartile is not concordant with the results in the other three quartiles. This finding raises additional questions about the reason why the insulin group has an increased rate of bone fractures. There are some plausible biological hypotheses. For instance, an increased number of hypoglycaemia readings was related with an increased risk of falls in the insulin group; however, it is possible that insulin therapy may exert its deleterious effects on bone tissue only at certain concentrations and/or that insulin at higher concentrations influences other intermediate factors affecting bone metabolism^29, 30^. The observational design and the aims of our study are not adequate to provide definitive answer to this key question.

The main limitation of our study was the lack of validation of each individual fracture. However, previous validation of fractures as recorded in SIDIAP has shown the database to be very precise (>95% accuracy for all fracture sites), compared with prospective cohort and hospital admissions data^41^. A recent validation study showed that the proposed list of ICD-10 codes is useful in distinguishing fragility (i.e., osteoporotic) fracture from high energy-induced fractures, with >90% of the coded hip fractures occurring after minimal trauma^42^.

The definition of insulin exposure used in the present study was based on pharmacy dispensation data. It is therefore reasonable to assert that patients used most of the dispensed doses; however, this could not be verified.

Another limitation was that the date of recorded T2DM diagnosis might not reflect the actual time of disease onset, which could have occurred months before a diagnosis was made and recorded.

One unresolved issue is the potential for residual confounding secondary to unobserved variables. Our database lacks variables such as BMD status, calcium/vitamin D or use of medication that may impact fracture risk, such as antidepressants, sedatives, and antipsychotics. However, this is unlikely to be imbalanced between treatment groups, as under current guidelines the indication for insulin therapy is based on T2DM metabolic control (such as HbA1c) and other parameters not including BMD or medications.

The misclassification of type 1 or low autoimmune diabetes of the adult (LADA diabetes) patients is also a potential limitation. Patients with T1DM or LADA improperly classified as T2DM require early insulin therapy initiation, which could explain the observed association with fracture risk. However, our sensitivity analysis excluding early insulin use (6 months post-T2DM diagnosis) did not support this explanation.

Finally, no pathophysiological information (circulating levels of insulin or other parameters potentially involved in bone health such as IGF-1, osteocalcin, and others) was available, nor were the number of hypoglycaemic readings, falls, menopause status, or specific fracture site. These data would be interesting; however, this information is usually not available in primary care databases.

Our study also has several notable strengths. It is the largest cohort study available on the effect of insulin use on fracture risk. The inclusion of only incident T2DM cases enables a more accurate assessment of the association with insulin use by reducing the effect of differential times from T2DM onset. The SIDIAP database contains detailed information on glycaemic control (HbA1c levels), not been accounted for in most previous studies. Additionally, our study uses state-of-the-art analytical methods (i.e., PS calliper matching) to minimize confounding via indication. Finally, the innovative use of a Fine and Gray survival analysis allowed us to adjust for competing risk with differential death according to insulin use.

The quality of the data used in this investigation has been confirmed in recent studies showing the representativeness of the SIDIAP database for the Catalan population^43–46^.

In conclusion, insulin therapy is related to approximately 40% increase in the risk of major fracture, even in early stages of T2DM. Assuming this association is causal, the use of insulin for 5 years in 82 patients with T2DM would induce 1 major osteoporotic fracture. These results suggest that the risk of fracture associated with insulin use should be taken into account in the process of deciding on a treatment approach; a systematic evaluation of fracture risk factors may be needed in all T2DM patients prior to the initiation of insulin treatment. Further studies, especially randomised controlled trials where possible, are needed to confirm this association.

## Research Design and Methods

### Data collection

This was a population-based matched cohort study of 53,853 newly diagnosed T2DM participants obtained from the SIDIAP database. SIDIAP contains primary care electronic medical records of a sample of patients in Catalonia (Northeast Spain), and includes a population of approximately 5 million patients (80% of the total population of Catalonia). Incorporated in the database, as part of routine practice, are the clinical and referral events registered by primary care administrative staff and health professionals (GPs and nurses), as well as demographic information, prescription and corresponding pharmacy invoicing data, specialist referrals, primary care laboratory test results, hospital admissions, and major patient outcomes.

We screened the SIDIAP database for patients who had an incident diagnosis of T2DM between 1 January 2006 and 31 December 2012. Exclusion criteria were a T2DM diagnosis date (i) before 1 January 2006 (i.e., pre-existing cases), (ii) before the registration date with the primary care practice or (iii) during the final year of the study period. Also excluded were users of diabetic medication longer than one month before T2DM diagnosis (likely pre-existing diabetes), insulin users with less than 2 months’ persistence (possible prescription errors), patients with advanced chronic kidney failure [estimated glomerular filtration rate (eGFR) ≤15], and patients <40 years of age on the date of T2DM diagnosis (potentially misclassified T1DM). No specific data collection procedures were applied. The index date was defined as 1 January 2006 or date of T2DM diagnosis (if after 1/1/2006). As data collection proceeded, each insulin user was PS-matched to 5 non-users, using data pertaining to the moment of T2DM diagnosis. Each patient was followed up from the T2DM diagnosis date to the first of the following end points: study end date, end of insulin treatment, fracture event, date transferred out of the practice, or death.

### Patient involvement

All data analysis was carried out in accordance with current and relevant guidelines and regulations. The study was approved by the Institut d´Investigació d´Atenció Primària Jordi Gol (IDIAP Jordi Gol). Active patient involvement activities and written informed consent were not required because all SIDIAP data were anonymized.

### Outcomes

The study outcome was an incident major bone fracture sustained after T2DM diagnosis and during the study period 1/1/2006 to 31/12/2013. Fracture events at the following sites were included as major fractures in the analyses: hip, clinical spine, pelvis, tibia, multiple rib, proximal humerus, and wrist/forearm. All fracture events were ascertained from primary care and hospital data using previously validated lists of ICD-10 codes^29, 30^.

### Exposures

The main exposure of interest was exposure to insulin for the treatment of T2DM, compared with no exposure to insulin. In secondary analyses, the exposures were the use of human vs. analogue insulin, long-acting vs. long-acting plus short-acting insulin and any insulin use with a high (third and fourth quartiles) MPR, compared to low MPR (first and second quartiles).

Long-acting human insulin (NPH), long-acting insulin analogues (NPL, glargine, or detemir insulin), short-acting insulins (regular, aspart, lispro, and glulisine), and premixed insulins (NPH plus regular insulin 70:30 or plus aspart insulin 30:70, 50:50, or 70:30; NPL plus lispro insulin 25:75 or 50:50) were considered.

Pharmacy dispensations of these medications were identified from the official regional reimbursement database (“Facturació de Farmàcia CatSalut” in Catalan) using national product codes, mapped to the WHO Anatomic Therapeutic Classification (ATC) codes and SIDIAP data. Treatment episodes were calculated as the time from the first to last insulin prescription, plus the number of daily-defined doses (DDDs as per the WHO ATC catalogue) purchased in the last prescription issued during the study period. For all comparisons, insulin use was defined as persisting two or more months.

### Confounders

Potential confounders of an insulin-fracture association were pre-defined based on clinical knowledge and a literature search for data pertaining to T2DM patients. These variables included age, gender, SES, calendar year of diagnosis, BMI, smoking status, and alcohol use. For BMI, smoking, and alcohol, only the value recorded nearest to T2DM diagnosis in the 5 years preceding the index date was used. We noted medications affecting fracture risk (steroids, diuretics, other anti-hypertensive drugs and mixed diuretics/anti-hypertensives) and osteoporosis treatment (parathyroid hormone, hormone replacement therapy, selective oestrogen receptor modulators (SERMs), and bisphosphonates). These medications were considered potential confounders in the 6 months prior to the index date. We also considered previous conditions affecting fracture risk (stroke, myocardial infarction, falls, and previous history of fracture) and diabetic complications (polyneuropathy, renal failure), HbA1c levels were considered in two different ways. As current HbA1c, the most recent value prior to T2DM diagnosis, and median HbA1c, the average of values during the follow up. We considered T2DM medications taken in the month prior to the index date such as metformin, sulphonylureas, meglitinides, dipeptidyl peptidase-4 inhibitors (i-DPP4), glucagon-like peptide-1 analogues (GLP-1) and other non-insulin medication for diabetes (alpha glucosidase inhibitors and thiazolidinediones).

### Statistical analysis

Due to the non-random allocation of insulin therapy, we calculated the PS for treatment with insulin, which was defined as a patient’s conditional probability of exposure to insulin given observed prognostic characteristics. We used multivariable logistic regression models to derive the score, including a priori confounders in addition to significant predictors (p < 0.2) of the outcome. Incident exposure to insulin (yes/no) was used as the binary outcome.

Confounding factors included in the final PS were those identified a priori or as predictive of outcome and were included in the model. Missingness in the above variables was incorporated as a separate category.

Using the R matching package, we matched each insulin user to 5 non-users within a specified calliper width of 0.2 of the standard deviation of the logit of the PS. The PS matching between insulin users and non-users was performed at the point of the T2DM diagnosis. The incidence rate of major osteoporotic fracture was estimated for insulin users and matched non-users. The first episode of continuous use of insulin (taking into account the switching of different insulin types) was considered, with the cessation of treatment defined as the last dispensation of insulin prior to a gap ≥6 months. Each patient was followed up from the diagnosis date of T2DM to the first of the following dates: end of study, end of insulin treatment, fracture event, transferred out, or death. A time-dependent covariate approach was taken, whereby follow-up for patients initiating insulin use after their diagnosis date for T2DM was split on the date of their first insulin dispensation to account for the switching of exposure status. In this manner, delayed users of insulin were considered non-users for the time period from their diagnosis date until their first dispensation date.

Univariable (matched) competing risk survival models (as proposed by Fine and Gray) were used, to account for the competing risk of death and to compare the time-to-fracture among insulin users *vs*. non-users^47^. The output of these models was SHRs, which are estimates of the relative instantaneous probability of fracture conditional upon survival (i.e., subjects are not removed from the sample when competing risks occur). Schoenfeld residuals from Cox models were used to check the validity of the proportional hazards assumption. Multivariable models were used to adjust for any confounding factors from the final PS model that remained unbalanced between the insulin users and non-users.

To explore the possibility of a dose-response relationship between insulin and fracture risk, we repeated the main analysis according to quartile of MPR for first insulin use. Here, we only considered the first insulin medication (censored upon switching) and recalculated the PS score, so matching was not identical, as in the main analysis, and the numbers of patients and fractures reported differed slightly. Furthermore, main analyses were repeated after multiple imputation with chained equations was used to impute missing values for BMI and HbA1c.

### Sensitivity analysis

We performed several sensitivity analyses to assess the robustness of our results using stratification and adjustment by PS. We studied the possible interactions of age, gender, and HbA1c levels in the primary outcome results by introducing multiplicative terms in the regression models. Insulin use was defined as patient retrieval of two months’ insulin prescription; to avoid immortal time bias, participants in the first two months of insulin treatment were considered as non-users (i.e., time-varying exposure) in a further sensitivity analysis^48^. To explore the potential impact of misclassification of T2DM on bone fracture, we also excluded the early insulin users (i.e., first 6 months after diagnosis), which permitted the exclusion of possible T1DM or LADA patients that may have been misclassified as T2DM.

A sensitivity analysis was also carried out to determine the impact of body mass on dose-effect, where the medication possession rate was divided by BMI and stratified by gender.

In a final sensitivity analysis, propensity-matched models were further adjusted for median HbA1c over follow up time.

## Electronic supplementary material

SUPPLEMENTARY TABLES
