# Supplementary material for: Insulin use and Excess Fracture Risk in Patients with Type 2 Diabetes: A Propensity-Matched cohort analysis
Source: Sci Rep. 2017 Jun 19;7:3781. doi: 10.1038/s41598-017-03748-z (PMC5476619; doi:10.1038/s41598-017-03748-z)
Supplement: Supplementary file 1 — SUPPLEMENTARY TABLES [file 41598_2017_3748_MOESM1_ESM.doc]

**TITLE PAGE**

**Insulin use and excess fracture risk in patients with type 2 diabetes: a propensity-matched cohort analysis**

Eladio Losada-Grande,MD ^(1)^ ^(2)^, Samuel Hawley, MSc^(3)^ , Berta Soldevila, MD, PhD ^(4)(5)^, Daniel Martinez-Laguna, MD^(6)^, Xavier Nogues,MD,PhD ^(7)(8)^, Adolfo Diez-Perez,MD, PhD ^(7)(8)^ Manel Puig-Domingo, MD,PhD ^(1)(4)(5)^, Dídac Mauricio,MD,PhD ^(4)(5)^ and Daniel Prieto-Alhambra, MD,MSc(Oxf),PhD ^(3)(6)^

^(1)^ Department of Medicine. Autonomous University of Barcelona. Barcelona, Spain

^(2)^ Endocrinology Section. Internal Medicine Department. Hospital Can Misses. Ibiza, Spain

^(3)^ Musculoskeletal Pharmaco- and Device Epidemiology, Centre for Statistics in Medicine. Nuffield Department of Orthopaedics. Rheumatology and Musculoskeletal Sciences. University of Oxford. Oxford, UK

^(4)^ Department of Endocrinology and Nutrition. University Hospital & Health Sciences Research Institute “Germans Trias i Pujol”. Badalona, Spain.

^(5)^ CIBER of Diabetes and Associated Metabolic Diseases (CIBERDEM). Instituto de Salud Carlos III, Spain.

^(6)^ GREMPAL Research Group. IDIAP Jordi Gol Primary Care Research Institute. Autonomous University of Barcelona. Barcelona, Spain

^(7)^ Internal Medicine Department. IMIM (Hospital del Mar Research Institute). Autonomous University of Barcelona. Barcelona, Spain.

^(8)^ CIBER of Healthy Ageing and Frailty Research (CIBERFes). Instituto de Salud Carlos III Spain.

**SUPPLEMENTARY TABLE 1**

**TABLE 1. Fracture incidence among matched users of any insulin vs non-users (with values imputed for BMI and HbA1c missingness )**

| Not on insulin (14,080) | | | On insulin (2,816) | | | SHR | adj SHR |
| --- | --- | --- | --- | --- | --- | --- | --- |
| N | median follow up (years) (IQR) | rate (per 1000 PYs) | N | median follow up (years) (IQR) | rate (per 1000 PYs) |
| 613 | 4.58 (2.60-6.32) | 8.60 (7.94- 9.30) | 57 | 1.42 (0.67-2.67) | 11.67 (9.00- 15.13) | 1.41 (1.08-1.86) | 1.44 (1.09-1.89) |

Only the first continuous insulin use is considered (switching types was accounted for), then censored. All analyses include incident insulin use as a time

Dependent covariate. Analyses are PS matched at a 5:1 ratio of non users to users. Adjusted analyses control for outstanding confounders (P<0.1).

Multiple imputation using chained equations was used for BMI and HbA1c.

Abbreviations: Body Mass Index (BMI), haemoglobin A1c (HbA1c), person-years (PYs), interquantile range (IQR), subhazard ratio (SHR), adjusted (adj)

**SUPPLEMENTARY TABLE 2**

**TABLE 2. Fracture incidence among matched users of any insulin vs non-users ( mean HbA1c adjusted )**

| Not on insulin (14,895) | | | On insulin (2,979) | | | SHR | adj SHR |
| --- | --- | --- | --- | --- | --- | --- | --- |
| N | median follow up (years) (IQR) | rate (per 1000 PYs) | N | Median follow up  (years) (IQR) | Rate (per 1000 PYs) |
| 631 | 4.58 (2.60-6.32) | 8.26 (7.64 - 8.93) | 60 | 1.42 (0.67-2.67) | 11.19 (8.69 - 14.42) | 1.53 (1.16-2.09); p=0.002 | 1.46 (1.11-1.91); p=0.007 |
| Only the first continuous insulin use considered (switching types was accounted for), then censored. All analyses include incident insulin use as a time dependent covariate. Analyses are PS matched, on a 5:1 ratio of non-users to users with mean Hba1c added as a covariate into final PS model. Adjusted analyses additionally control for outstanding confounders (P<0.1), i.e. BMI, steroids, meglitinides and anti-hypertensives. | | | | | | | |

**SUPPLEMENTARY TABLE 3**

**TABLE 3. Fracture incidence among matched users of any insulin vs non-users stratified by quartiles of insulin medication possession ratio (MPR) / body mass index (BMI) in men.**

| MPR/BMI | | Not on insulin (4,742) | | | On insulin (1,650) | | | SHR | adj SHR |
| --- | --- | --- | --- | --- | --- | --- | --- | --- | --- |
| quartile | median (IQR) | N | median follow up (years) | rate (per 1000 PYs) | N | median follow up (years) | rate (per 1000 PYs) |
| 1 | 0.70 (0.53-0.81) | 36 | 4.08 (1.93-6.13) | 5.72 (4.13-7.94) | 2 | 0.67 (0.25-2.17) | 3.45 (0.86-13.80) | 0.65 (0.15-2.69) | 0.60 (0.14-2.54) |
| 2 | 1.24 (1.10-1.40) | 14 | 4.44 (2.37-6.15) | 2.14 (1.27-3.61) | 4 | 0.92 (0.50-2.09) | 6.50 (2.44-17.32) | 3.03 (0.99-9.28) | 2.97 (0.91-9.66) |
| 3 | 1.94 (1.77-2.17) | 27 | 4.36 (2.22-6.15) | 4.19 (2.87-6.11) | 10 | 1.59 (0.92-2.75) | 12.66 (6.81-24.53) | 3.21 (1.55-6.66) | 3.00 (1.43-6.30) |
| 4 | 3.22 (2.74-3.96) | 44 | 4.50 (2.25-6.34) | 6.62 (4.92-8.89) | 4 | 2.08 (1.17-3.26) | 4.16 (1.56-11.08) | 0.64 (0.23-1.78) | 0.59 (0.22-1.60) |
| Only the first insulin use considered (switching types was not accounted for), then censored. All analyses include incident insulin use as a time dependent covariate. Analyses are PS matched, on a 5:1 ratio of non-users to users. Adjusted analyses control for outstanding confounders (P<0.1) and for propensity score. Multiple imputation using chained equations was used to impute values where BMI was missing. Final PS model restricted to males (if final matched sets had no males or comparators then they were also dropped) | | | | | | | | | |

**SUPPLEMENTARY TABLE 4**

**TABLE 4. Fracture incidence among matched users of any insulin vs non-users stratified by quartiles of insulin medication possession ratio (MPR) / body mass index (BMI) in women.**

| MPR/BMI | | Not on insulin (2,899) | | | On insulin (1,228) | | | SHR | adj SHR |
| --- | --- | --- | --- | --- | --- | --- | --- | --- | --- |
| quartile | median (IQR) | N | median follow up (years) | rate (per 1000 PYs) | N | median follow up (years) | rate (per 1000 PYs) |
| 1 | 0.65 (0.51-0.77) | 58 | 3.97 (1.90-6.00) | 14.80 (11.44-19.14) | 5 | 0.75 (0.33-1.92) | 13.16 (5.48-31.61) | 0.93 (0.37-2.35) | 0.94 (0.37-2.38) |
| 2 | 1.23 (1.07-1.36) | 53 | 4.33 (2.06-6.21) | 12.89 (9.85-16.88) | 6 | 1.00 (0.42-2.08) | 14.26 (6.41-31.74) | 1.14 (0.49-2.61) | 1.05 (0.45-2.45) |
| 3 | 1.84 (1.64-2.07) | 45 | 3.91 (1.94-5.81) | 11.74 (8.76-15.72) | 9 | 1.67 (0.83-2.75) | 15.38 (8.00-29.55) | 1.26 (0.61-2.58) | 1.20 (0.58-2.50) |
| 4 | 3.05 (2.56-3.71) | 57 | 3.94 (1.83-5.86) | 14.28 (11.01-18.51) | 14 | 1.76 (1.09-2.67) | 23.08 (13.67-38.98) | 1.73 (0.95-3.14) | 1.73 (0.95-3.15) |
| Only the first insulin use considered (switching types was not accounted for), then censored. All analyses include incident insulin use as a time dependent covariate. Analyses are PS matched, on a 5:1 ratio of non-users to users. Adjusted analyses control for outstanding confounders (P<0.1) and for propensity score. Multiple imputation using chained equations was used to impute values where BMI was missing. Final PS model restricted to females (if final matched sets had no females or comparators then they were also dropped) | | | | | | | | | |
